# Changes in bacterial communities during rice cultivation remove phenolic constraints on peatland carbon preservation

**DOI:** 10.1093/ismeco/ycae022

**Published:** 2024-02-06

**Authors:** Lei Qin, Wei Tian, Chris Freeman, Zhongjun Jia, Xiaolei Yin, Chuanyu Gao, Yuanchun Zou, Ming Jiang

**Affiliations:** State Key Laboratory of Black Soils Conservation and Utilization, Key Laboratory of Wetland Ecology and Environment, Heilongjiang Xingkai Lake Wetland Ecosystem National Observation and Research Station, Northeast Institute of Geography and Agroecology, Chinese Academy of Sciences, Changchun 130102, China; College of Forestry and Grassland, Jilin Agriculture University, Changchun 130118, China; School of Natural Sciences, Bangor University, Bangor LL57 2UW, United Kingdom; State Key Laboratory of Black Soils Conservation and Utilization, Key Laboratory of Wetland Ecology and Environment, Heilongjiang Xingkai Lake Wetland Ecosystem National Observation and Research Station, Northeast Institute of Geography and Agroecology, Chinese Academy of Sciences, Changchun 130102, China; State Key Laboratory of Black Soils Conservation and Utilization, Key Laboratory of Wetland Ecology and Environment, Heilongjiang Xingkai Lake Wetland Ecosystem National Observation and Research Station, Northeast Institute of Geography and Agroecology, Chinese Academy of Sciences, Changchun 130102, China; State Key Laboratory of Black Soils Conservation and Utilization, Key Laboratory of Wetland Ecology and Environment, Heilongjiang Xingkai Lake Wetland Ecosystem National Observation and Research Station, Northeast Institute of Geography and Agroecology, Chinese Academy of Sciences, Changchun 130102, China; State Key Laboratory of Black Soils Conservation and Utilization, Key Laboratory of Wetland Ecology and Environment, Heilongjiang Xingkai Lake Wetland Ecosystem National Observation and Research Station, Northeast Institute of Geography and Agroecology, Chinese Academy of Sciences, Changchun 130102, China; State Key Laboratory of Black Soils Conservation and Utilization, Key Laboratory of Wetland Ecology and Environment, Heilongjiang Xingkai Lake Wetland Ecosystem National Observation and Research Station, Northeast Institute of Geography and Agroecology, Chinese Academy of Sciences, Changchun 130102, China

**Keywords:** keystone bacterial taxa, phenolic inhibition, carbohydrates, carbon mineralization rate, cultivated peatlands

## Abstract

Northern peatlands contain ~30% of terrestrial carbon (C) stores, but in recent decades, 14% to 20% of the stored C has been lost because of conversion of the peatland to cropland. Microorganisms are widely acknowledged as primary decomposers, but the keystone taxa within the bacterial community regulating C loss from cultivated peatlands remain largely unknown. In this study, we investigated the bacterial taxa driving peat C mineralization during rice cultivation. Cultivation significantly decreased concentrations of soil organic C, dissolved organic C (DOC), carbohydrates, and phenolics but increased C mineralization rate (CMR). Consistent with the classic theory that phenolic inhibition creates a “latch” that reduces peat C decomposition, phenolics were highly negatively correlated with CMR in cultivated peatlands, indicating that elimination of inhibitory phenolics can accelerate soil C mineralization. Bacterial communities were significantly different following peatland cultivation, and co-occurrence diagnosis analysis revealed substantial changes in network clusters of closely connected nodes (modules) and bacterial keystone taxa. Specifically, in cultivated peatlands, bacterial modules were significantly negatively correlated with phenolics, carbohydrates, and DOC. While keystone taxa *Xanthomonadales*, *Arthrobacter*, and *Bacteroidetes_vadinHA17* can regulate bacterial modules and promote carbon mineralization. Those observations indicated that changes in bacterial modules can promote phenolic decomposition and eliminate phenolic inhibition of labile C decomposition, thus accelerating soil organic C loss during rice cultivation. Overall, the study provides deeper insights into microbe-driven peat C loss during rice cultivation and highlights the crucial role of keystone bacterial taxa in the removal of phenolic constraints on peat C preservation.

## Introduction

Northern peatlands cover only 3% of global land area but store up to 540 Pg of carbon (C), thereby sequestering ~30% of the global terrestrial C pool [1, 2]. However, 14% to 20% of peatlands have been drained and cultivated globally to meet production demands [3]. It is estimated that this cultivation resulted in 40 Pg of C emissions since 1750, accounting for 67% of land-use C emissions in the Northern Hemisphere [4]. Paddy management with irrigation in the growing season can slow peat-derived C emissions, and thus, irrigation represents a relatively optimized pattern in mitigating C emissions compared with other management practices, such as those for upland crops and pasture [5, 6]. However, with increasing human intervention (notably fertilization), C stability remains under threat [7]. Microorganisms are recognized as primary decomposers, and previous studies show dramatic changes in microbial activity and diversity during peatland cultivation [8-10]. However, the precise influence of the microbial community on C decomposition remains poorly understood.

Dissolved organic carbon (DOC) is an important fraction of the C pool and represents a pivotal component modulating soil biogeochemical processes [11, 12]. Dissolved organic C production and variations in its chemical components can determine soil C turnover by influencing soil microbial respiration [13-15]. Phenolics, characterized by one or more aromatic rings bearing one or more hydroxyl functional groups [16], represent critical components of the DOC fractions and are widely reported to constrain organic C decomposition in peatlands [17, 18]. The critical mechanism of phenolic action involves deactivation of enzyme activity and direct toxicity on microbes [17, 19]. In peatlands, waterlogged conditions limit phenol oxidative activity and lead to phenolic accumulation, thereby forming an inhibitory “phenolic latch” on C decomposition [17]. In addition to peatlands, phenolic compounds also inhibit soil respiration in agricultural, aquatic, and forest ecosystems [20-23]. Previous studies show that peatland cultivation can significantly decrease phenolic content [22, 24], which could potentially remove phenolic inhibition of microbial activity and accelerate microbially driven C decomposition.

Fungi, especially ectomycorrhizal and white rot fungi, can degrade phenolic compounds by producing oxidative extracellular enzymes [25]. However, fungi are generally adapted to aerobic conditions, and their oxidative enzymes require oxygen or oxygen-generated hydrogen peroxide to function [26]. Therefore, fungi exert only weak effects on C mineralization under permanent and periodically flooded conditions [27, 28] and thus are unlikely to explain the changes in C mineralization rate between natural and rice-cultivated peatlands. By contrast, bacteria-mediated phenolic degradation of polyphenols under anoxia is highly effective [29, 30], with the taxa involved including methanogens, denitrifiers, and iron-reducing and sulfate-reducing bacteria [31, 32]. Zwetsloot *et al.* [33] showed that different phenolic compounds (benzoic acid, caffeic acid, and catechin) caused different degrees of inhibition of bacterial activity and abundance, whereas others, such as benzoic acid in glucose-amended soils, stimulated organic C decomposition by changing the bacterial community. Nutrient addition can accelerate C decomposition by alleviating microbial nutrient limitations and increasing the oxidation of phenolics [18, 34]. Although many members of the bacterial community can potentially degrade phenolics, wider substrate availability can often constrain the activity of those bacteria.

Microbial communities with similar functional traits (e.g. roles in nitrogen and phosphorus cycling and organic matter decomposition) often show similar adaptations to environmental change [35]. Agricultural reclamation of peatlands changes both flooding and nutrient regimes, which influences the abundance and composition of phenolic-degrading members of the microbiota and thus C mineralization. Most studies focus only on testing the effects of microbial biomass, activities, or community diversity on soil C mineralization [36] and therefore lack a holistic understanding of microbial functioning and community-mediated C loss during peatland cultivation. Recently, microbial co-occurrence networks have been widely applied to analyse microbial community structure and functional groups [37-39]. Importantly, network scores can be used to statistically identify keystone taxa, which are highly connected taxa that individually or in a guild, exert a significant influence on community structure and function irrespective of their abundance across space and time [40]. Culturing experiments have verified the importance of keystone taxa identified by network analysis [41]. Therefore, identifying keystone taxa and associated functional groups can increase understanding of soil biogeochemistry during peatland cultivation.

To investigate the dynamics of bacterial communities in promoting peat C mineralization during rice cultivation, we selected three pairs of typical peatlands, each of which contained both natural and rice-cultivated peatlands, in Northeast China. How changes in bacterial communities affected the soil organic C mineralization rate was examined in both natural and rice-cultivated peatlands, and the keystone taxa associated with those processes were identified. We hypothesized that (i) the resultant decreases in inhibitory phenolics during peatland cultivation would increase soil C mineralization rate and that (ii) keystone taxa would strongly influence the bacterial community associated with phenolics degradation in rice-cultivated peatlands.

## Materials and methods

### Study site and sampling

**Figure 1:**
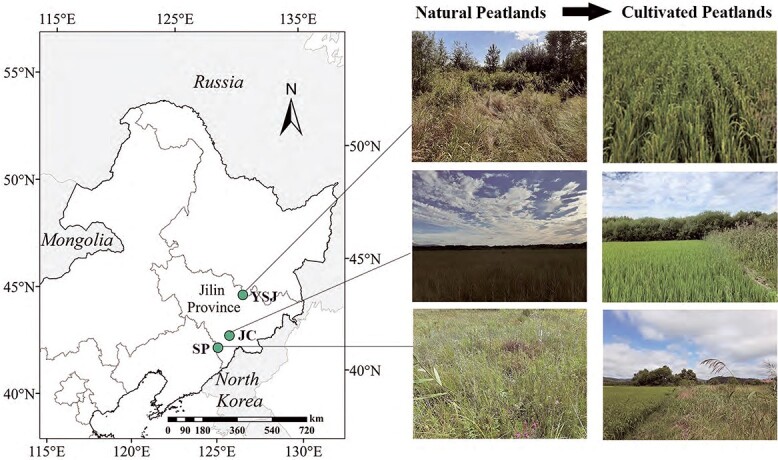
Study sites containing natural and cultivated peatlands in Jinchuan (JC), Sipeng (SP), and Yushugou (YSJ) peatlands in Northeast China.

Northeast China contains abundant peatlands, accounting for 48% of the total wetland area in China [42]. To meet the demand for food, over half of the peatlands were reclaimed as croplands between 1960 and 2000 [43]. We identified three typical peatlands (Fig. 1) in which part had been cultivated for rice for >20 years: Sipeng, Jinchuan, and Yushugou peatlands. Before the 1980s, the Sipeng peatland (SP) (41°51′29″N, 125°34′48″E) encompassed ~40 ha, the peat thickness was 0.8–1.2 m, and the dominant species was *Carex schmidtii*. In the early 2000s, ~90% of the SP was cultivated as paddy fields, dry croplands, or fishponds. The Yushugou peatland (YSJ) (44°09′00″N, 127°31′12″E) is a typical shrub-dominated peatland, with peat thickness of ~1.0 m. Part of YSJ has been cultivated for rice since the 1970s. The Jichuan peatland (JC) (42°21′00″N, 126°22′12″E) was ~100 ha, with peat thickness 0.8–3.0 m and the dominant plant *C. schmidtii*. Rice cultivation in JC began in 1960; however, many sites were tilled for maize and ginseng, and the remnant rice-cultivated peatlands were only cultivated for ~20 years. In general, the paddy fields were irrigated in May, drained at the end of September, and harvested in October. Nitrogen fertilizer (urea) was applied mid-May and throughout June for rice growth, for a total of 260 kg N ha^−1^ year^−1^. Phosphorus fertilizer was applied in mid-May only at 70 kg P ha^−1^ year^−1^.

To test the association between the bacterial community and peat C mineralization under flooded conditions during rice cultivation, samples were collected from both natural and adjacent cultivated peatlands in the three sites in the middle of August 2021, when the time of greatest biological influence on soil processes. Four independent 2 m × 2 m plots were selected within each type (separated by >50 m). After removing aboveground biomass and litter, five subplots were set up within each plot. Subplot soils at 0–15 cm and 15–30 cm were collected by Russian peat corer (5 cm in diameter) and then mixed into a composite sample, resulting in 48 samples (3 sites × 2 types × four plots × 2 depths). Soil samples were divided into two parts. One part was stored at 4°C and used to analyse soil C mineralization rate. The other part was stored at −80°C and then freeze-dried to preserve the integrity of nucleic acids and to improve the homogenization of samples for study of the bacterial community [44]. After freeze-drying, samples were immediately extracted for DNA. The remnant freeze-dried samples were subjected to further grinding before soil C chemistry and chemical indicators were measured.

### Sample analysis

#### Soil organic matter chemistry

To determine soil organic C (SOC) content, soil carbonate was first removed by 1 M HCl, and then, samples were freeze-dried. Organic C content was measured by an elemental analyzer (Elemental Vario EL, Hanau, Germany).

Fourier-transformed infrared spectroscopy (FTIR) was used to indicate the recalcitrance of soil C with a spectrometer (Bruker Tensor-27, Ettlingen, Germany), which has a range of 4000–400 cm^−1^ at a 4 cm^−1^ resolution (Fig. S1). Specifically, polysaccharides were represented by an absorption band at ~1033 cm^−1^, whereas recalcitrant C (i.e. lignin and other aromatics and aromatic or aliphatic carboxylates) were indicated by an absorption band at ~1630 cm^−1^ (Broder et al., 2012). Corrected peak area ratios of 1630/1030 were calculated to estimate the relative abundance of recalcitrant to labile C in soil [45].

Soil DOC was extracted from a soil suspension (soil to water ratio = 1:20) by shaking for 12 h. Suspensions were filtered through precombusted 0.45-mm filters. The concentration of DOC was measured by a multi N/C 3100 total organic carbon analyzer (Analytik-jena, Jena, Germany). Filtered suspensions were also measured for phenolic compounds by the Folin–Ciocalteau method [17]. The remnant suspensions were freeze-dried before estimating the carbohydrate contents of the DOC using FTIR spectra. Using the same method as for solid sample FTIR absorbance measurements, the carbohydrate content (%) was estimated by the relation between area-normalized peak height (carb, ~1030 cm^−1^) and contents of cellulose + hemicellulose (carbohydrate contents = 49 204 × carb −1.7606) [46]. The carbohydrate content of the DOC was further calculated according to the proportion of carbohydrates in each sample.

#### Soil nutrient levels

Soil total nitrogen (TN) content was determined by elemental analyzer (Elemental Vario EL). To determine total phosphorus (TP), soils were digested by nitric and perchloric acids. To determine available phosphorus (AP), soils were extracted using 0.5 mol L^−1^ NaHCO_3_. To determine available nitrogen (AN), soils were extracted by 2 M KCl. Finally, extracted P and N in suspensions were measured by a continuous flow analyzer (Skalar SAN^++^, Breda, Netherlands). Soil pH was determined using a pH meter at a soil to water ratio of 1:5 (Leici PHS-3E, Shanghai, China).

#### Soil carbon mineralization potential

To determine soil C mineralization rate (CMR), 30 g of fresh peat sample from each plot was added to flasks (250 ml). Flasks were sealed with rubber stoppers and incubated at 20°C for 10 h. Gas samples were collected at 0 (T0) and 10 h (T1), and CO_2_ concentration was determined by gas chromatograph (Agilent 7890B, Santa Clara, CA, USA) as C0 and C1, respectively. The difference in CO_2_ concentration between T0 and T1 was used to estimate the rate of C mineralization. Given the difference in initial soil organic C, the mineralization rate was further normalized by the soil organic C [47], such that the rate was calculated as follows:


$$ \mathrm{CMR}={\Delta }_c\times V\times M/\left(m\times{\Delta }_t\times 22.4\right)\times \mathrm{\alpha} /\mathrm{SOC} $$

where CMR is the production rate of each treatment (mg C • kg^−1^ SOC• d^−1^); V is the void volume of the incubation bottle (L); M is C molar mass of CO_2_; m is the weight of soil (kg);${\Delta }_c$is the ratio of change in the gas concentrations (C1–C0); ${\Delta }_t$ is the incubation time (days); 22.4 is the molar volume of CO_2_ in the standard conditions (L mol^−1^); α is a conversion factor for temperature 273/(273 + T), and T is the incubation temperature; and SOC is the soil organic C.

#### DNA extraction, 16S rRNA gene sequencing, and bioinformatics analyses

Soil DNA was extracted using a Power Soil Isolation kit (MoBio Laboratories, Carlsbad, CA, USA). After evaluation of DNA quality by a NanoDrop 2000 spectrophotometer (Thermo Fisher Scientific, Cleveland, OH, USA), bacterial 16S rRNA V4 was amplified using primers 515F and 806R. Amplicon sequences were analyzed on an Illumina Novaseq platform (Illumina, San Diego, CA, USA). The 16S rRNA gene sequences were processed using QIIME v2 (Quantitative Insights into Microbial Ecology, https://qiime2.org). First, paired-end Illumina reads were processed by merging, removing bar codes and primers, and filtering low-quality reads. Second, the sequence reads were clustered into operational taxonomic units (OTUs) with 97% similarity. All samples were rarefied to 39 575 sequences per sample and had an average of 8106 OTUs. The bacterial OTUs were annotated according to the SILVA 132 database (www.arb-silva.de/documentation/release-132/). After removing the 202 archaea taxa OTUs that accounted for an average of 4.3% of the total sequences across the samples, 7904 bacterial OTUs per sample were ultimately obtained.

### Statistical analyses

Mixed-effect models were used to assess the effects of cultivation and soil depth on soil organic C, C chemistry, C mineralization potential, bacterial diversity, and phyla-level taxa using the “lme” function in the R package “nlme” in R v3.6.2 (R Core Team, 2019). In the models, “soil depth in natural and cultivated peatlands” was set as a fixed effect, and “site” was defined as a random effect. When there was a significant effect, a multiple comparison test (Tukey’s HSD) was conducted to test the differences in variables with the “multcomp” package in R.

To analyze bacterial communities, alpha diversity at the species level was determined by richness and Shannon indices. Nonmetric multidimensional scaling (NMDS) and community similarity was analyzed by the functions of metaMDS and anosim in the “vegan” package in R v3.6.2, and ordination “stress” was used to evaluate the fit of an NMDS ordination. Generally, a stress value <0.2 indicates that the ordination varies from the original sample and can be used for interpretation.

To identify keystone bacterial taxa, bacterial association networks were created by using the most abundant 2000 OTUs, which accounted for more than 70% of bacteria. The correlation matrix was generated by the SparCC method [48]. The network matrix was filtered under a threshold value of 0.6 and a significance level of 0.05. Nodes in the network indicated bacterial taxa (OTUs), and modules were clusters of closely connected nodes. The node connection parameter within a module (Zi) and the connectivity of modules parameter (Pi) were selected to estimate the critical role of nodes, including module hubs (Zi > 2.5, Pi <0.625), connectors connected modules (Zi < 2.5, Pi >0.625), and network hubs (Zi > 2.5, Pi >0.625) [49]. In addition, the first principal component of module data (module eigengene) was calculated for the keystone gene analysis, which represented connected members within a module [50]. The above analysis was conducted using the Inter-Domain Ecological Network Analysis Pipeline (http://mem.rcees.ac.cn:8081) [51]. Hub genes regulating C mineralization were identified according to the association of genes with a module eigengene (module membership, MM) and the association of genes with CME (gene significance, GS). Hub genes were defined according to the significance level of 0.05 for both GS and MM. The keystone taxa associated with C mineralization were identified by the following criteria: 1) the bacterial taxa belonged to the hub genes or critical nodes (module hubs, connectors, or network hubs) and 2) bacterial taxa have C metabolism functions. Gene analysis was conducted using the R package “WGCNA” in R v3.6.2 [52].

Pearson correlation tests were conducted to test the effects of phenolics and carbohydrates on soil mineralization, as well as the effects of keystone taxa on CMR, with significance set at the 0.05 level.

## Results

### Soil organic carbon, carbon chemistry, soil carbon mineralization, and soil properties

**Figure 2:**
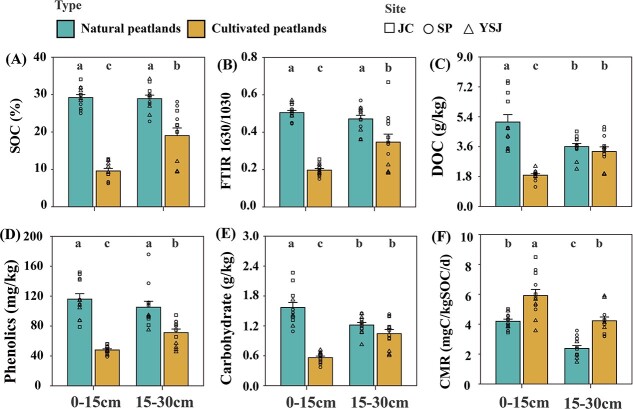
Effects of cultivation and depth on soil organic carbon (C), C chemistry, and C mineralization. JC, Jiangchuan; SP, Sipeng; YSJ, Yushugou; SOC, soil organic C; soil recalcitrant index (FTIR 1630/1030); DOC, dissolved organic C; CMR, C mineralization rate. Values are the mean ± SE, *n* = 12. Different letters indicate significant differences (*P* < 0.05).

Soil organic C, FTIR 1630/1030 ratio, and phenolics contents showed no differences between surface (0–15 cm) and subsurface (15–30 cm) soils, whereas DOC and carbohydrate content were significantly higher in surface soil than in subsoil in natural peatlands (Fig. 2A-E). Cultivation significantly decreased SOC, FTIR 1630/1030 ratio, and phenolics content of both surface and subsurface soils (*P* < 0.01, Fig. 2A-E), and the average loss in those indices was greater in surface soil than in subsoil. Cultivation only reduced DOC and carbohydrates in surface soils (*P* < 0.05, Fig. 2C and E). Notably, CMR in surface and subsurface soils of cultivated peatlands was significantly higher than that in natural peatlands (*P* < 0.001, Fig. 2F). In addition, cultivation significantly decreased TN and increased TP, AP, and pH in both surface and subsurface soils (*P* < 0.05), whereas it only decreased AN in surface soil (*P* < 0.05, Tables S1–2).

**Figure 3:**
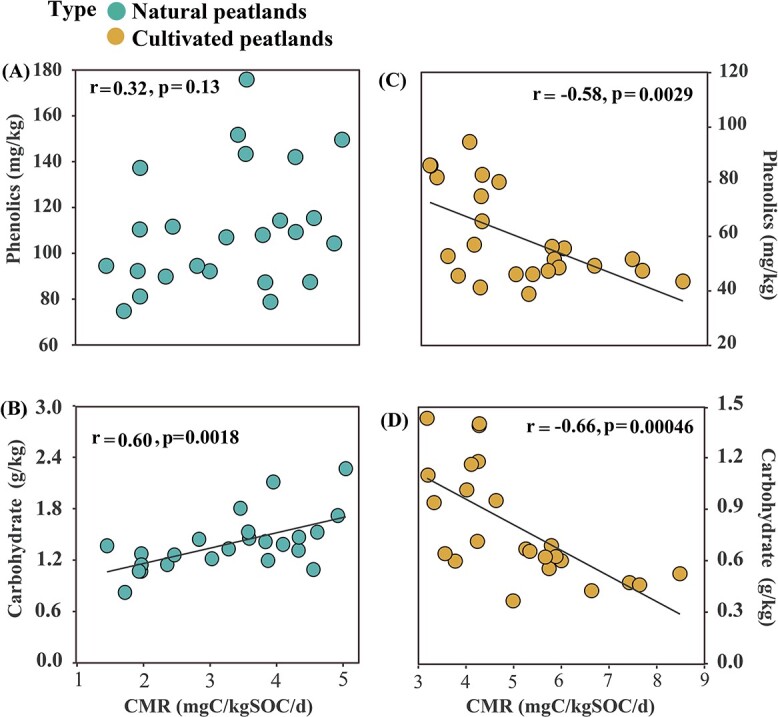
Correlations between carbohydrates and phenolics and carbon mineralization rate (CMR). Correlations between (A) CMR and phenolics in natural peatlands; (B) CMR and carbohydrates in natural peatlands; (C) phenolics and CMR in cultivated peatlands; and (D) CMR and carbohydrates in cultivated peatlands. Solid lines indicate significant linear correlations.

Relations between dissolved labile (carbohydrates) and recalcitrant C (phenolic compounds) and CMR in natural and cultivated peatlands were examined. Only carbohydrate content was positively correlated with CMR in natural peatlands (*r* = 0.60, *P* = 0.0018; Fig. 3B). However, both phenolics and carbohydrate contents were significantly negatively correlated with CMR in cultivated peatlands (phenolics: *r* = −0.58, *P* = 0.0029, Fig. 3C; carbohydrates: *r* = −0.66, *P* = 0.00046, Fig. 3D).

### Soil bacterial communities

**Figure 4:**
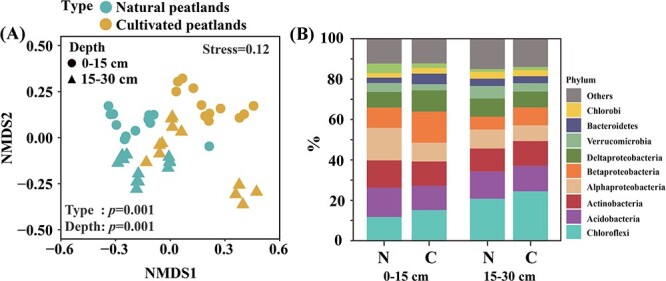
Bacterial community composition in natural and cultivated peatlands. (A) Nonmetric multidimensional scaling (NMDS) of bacterial community composition. The stress value <0.2 indicates that the ordination varied from the original sample and that it is useful for interpretation, and the significance of differences between natural and cultivated peatlands or between surface and subsurface soils was tested by analysis of similarities. (B) Comparisons at the phylum level across depths during peatland cultivation. Values are the mean ± SE, *n* = 12.

Cultivation did not affect richness and Shannon diversity indices of bacterial communities (Fig. S2A and B). However, in the NMDS analysis and differences analysis at the phylum- level, both cultivation and soil depth significantly altered soil bacterial community composition (Fig. 4A and B and Table S3).

**Figure 5:**
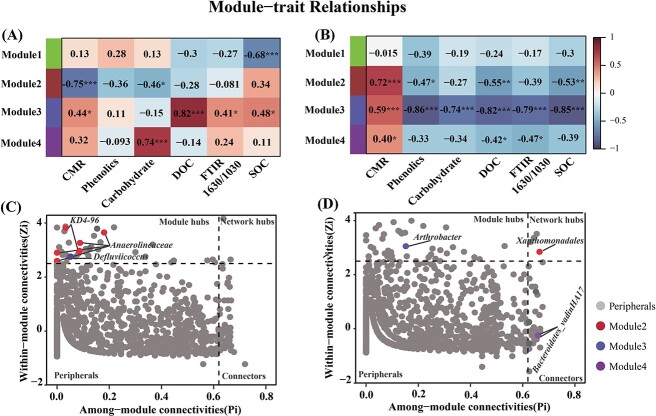
Correlation coefficients for module eigengenes with carbon mineralization rate (CMR), phenolics, carbohydrates, dissolved organic C (DOC), FTIR 1630/1030 ratio, and soil organic C (SOC) in (A) natural and (B) cultivated peatlands. ^*^P < 0.05; ^**^P < 0.01; ^***^P < 0.001. Distribution of bacterial keystone taxa related to CMR in (C) natural and (D) cultivated peatlands. Dashed lines represent threshold values of the node connection parameter within module (Zi) and the connectivity of the modules parameter (pi) for categorizing species.

Bacterial networks were constructed to assess changes in bacterial communities associated with peatland cultivation. The topological characteristics of number of edges, number of positive edges, number of negative edges, and modules in co-occurrence networks of bacteria differed between natural and cultivated peatlands (Table S4 and Fig. S2). Networks were examined to determine the significant module–trait relations for CMR and C fractions in natural and cultivated peatlands (Fig. 5A and B). In natural peatlands, the eigengene of module#2 was significantly negatively correlated with CMR and carbohydrate content, whereas the eigengene of module#3 was significantly positively correlated with CMR, DOC, FTIR 1630/1030 ratio, and SOC (*P* < 0.001, Fig. 5A). By contrast, in the cultivated peatland, the eigengene of module#2, module#3, and module#4 was significantly positively correlated with CMR (*P* < 0.001, Fig. 5B). Module#2 was negatively correlated with phenolics, DOC, and SOC; module#3 was negatively correlated with phenolics, carbohydrates, DOC, FTIR 1630/1030 ratio, and SOC; and module#4 was negatively correlated with DOC and FTIR 1630/1030 ratio (Fig. 5B).

In natural peatlands, module–trait relations for soil variables indicated that the eigengene of module#2 was significantly negatively correlated with soil AP, and that the eigengene of module#3 was significantly positively correlated with soil AN and AP (Fig. S4A). In cultivated peatlands, the eigengene of module#2 was significantly negatively correlated with TN and AN but significantly positively correlated with pH, and the eigengene of module#3 was significantly positively correlated with TP and AP but significantly negatively correlated with TN. Similarly, the eigengene of module#4 was significantly negatively correlated with TN and AN (Fig. S4B).

### Keystone taxa associated with soil carbon loss

**Figure 6:**
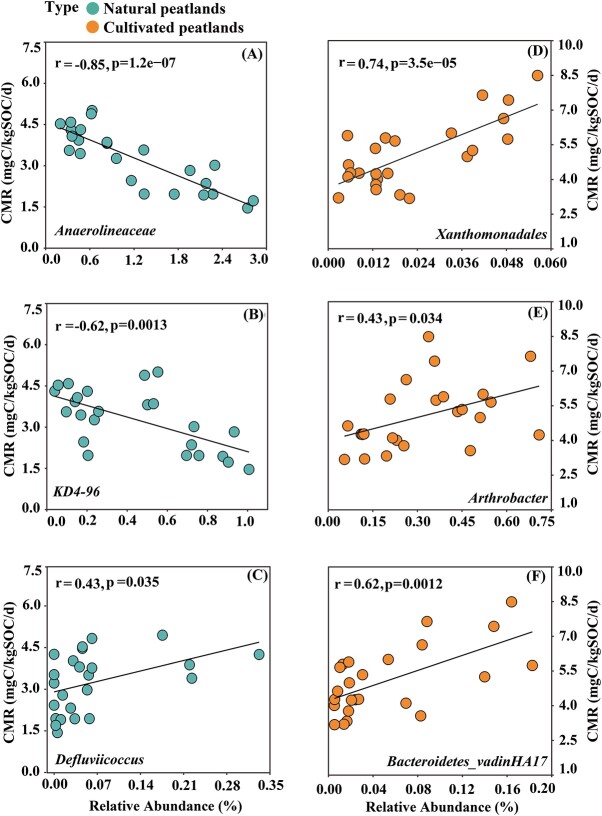
Relations between bacterial keystone taxa and carbon mineralization rate (CMR) in (A, B, C) natural and (D, E, F) cultivated peatlands. SOC: Soil organic carbon. Solid lines indicate significant linear correlations.

Keystone genes associated with C dynamics were identified from the nodes of module hubs, connectors and network hub nodes by using gene significance (GS) and module membership (MM) (Table S5 and Fig. 5C and D). In natural peatlands, *Anaerolineaceae* and *KD4–96* were the keystone taxa that negatively correlated with CMR, whereas the keystone taxon *Defluviicoccus* was positively correlated with CMR (Fig. 6A–C). In cultivated peatlands, *Arthrobacter*, *Xanthomonadales*, and *Bacteroidetes_vadinHA17* were the keystone taxa positively correlated with CMR (Fig. 6D–F).

## Discussion

### Carbon fractions and their influence on carbon mineralization

Cultivation significantly decreased SOC content, consistent with previous studies [4], and also FTIR 1630/1030 ratio, carbohydrates, DOC, and phenolics (Fig. 2A–D). The FTIR band at ~1630 cm^−1^ represents lignin and other aromatics and aromatic or aliphatic carboxylates [53], and those complex macromolecules are recalcitrant and resist degradation, with only strong oxidants such as phenol oxidase and peroxidase extracellular enzymes able to break down the complex lignin structures [54]. In peatlands, anoxic or hypoxic conditions limit abiotic and biotic oxidative processes, promoting the accumulation of plant-derived lignin and phenolics [17]. In natural peatlands, both DOC and carbohydrates were higher in surface than in subsurface soils (Fig. 2C and E). This result is mainly because plant litter and root exudates dominate in surface soils and contain large and labile forms of C [54], which contribute to greater carbohydrate accumulation in surface than in subsurface soils. In cultivated peatlands, losses in SOC, DOC, carbohydrates, and phenolics and decreases in the FTIR 1630/1030 ratio were greater in surface than in subsurface soils (Fig. 2A–D). Agricultural practices including fertilization and irrigation management, oxygen secretion by rice roots, water level fluctuations, and changes in temperature are all greater in surface soils than in subsurface soils [54], which also accelerate microbial C mineralization. In addition, the loss of SOC and decreases in FTIR 1630/1030 ratio in subsurface soils of the long-term cultivated site (YSJ) were greater than those in JC and SP (Fig. 2A and B). Rice has been cultivated in YSJ for ~50 years, whereas in JC and SP, rice has only been cultivated for 20 years, suggesting that the number of years of cultivation is an important factor driving C loss during peatland cultivation.

The effects of phenolics on CMR were also evaluated; however, no relations with CMR were identified in natural peatlands (Fig. 3A). Although phenolics are toxic and can inhibit microbial heterotrophic respiration [17, 18], some positive and neutral relations between phenolics and C mineralization have been observed [33, 55]. Two key factors influence phenolic inhibition of C mineralization: (i) the molecular structure of phenolics and the notably relatively high molecular weight of many phenolics, which tend to inhibit microbial activity [56] and (ii) abundant labile C, which has stimulatory effects that can offset the inhibitory effects of phenolics in DOC [57], thereby counteracting the net inhibitory effect on microorganisms. In this study, the surface labile C (carbohydrate content) of natural peatlands was more than twice that of cultivated peatlands (Fig. 2E), and carbohydrate content was positively correlated with CMR (Fig. 3B), although there was no apparent relation between phenolics and DOC in the natural peatland (Fig. S3A). Those observations suggest that in natural sites, abundant labile C is important in counteracting phenolic inhibition of C mineralization. By contrast, the observation that phenolics were negatively correlated with CMR (Fig. 3C), supports the hypothesis that decreases in inhibitory phenolics during peatland cultivation promotes soil C mineralization. Increases in nutrient and oxygen availability during wetland cultivation promote microbial decomposition of labile C, whereas microorganisms are unlikely to utilize recalcitrant C until labile substrates are depleted [58]. In this study, cultivation significantly decreased carbohydrates in DOC (Fig. 2E), whereas phenolics and DOC were significantly positively correlated (Fig. S3B). However, after the degradation of much of the labile C that occurs during rice cultivation, the “labile C offset effect” found in natural peatland would be reduced. Ultimately, phenolics appear to play a dominant role in inhibiting C mineralization in peatlands that have been cultivated over decadal time scales. In addition, phenolics degradation during peatland cultivation further removes phenolic constraints on microbial activity and enzyme activities, promoting additional decomposition of labile C [17]. This result is consistent with the finding that carbohydrates were negatively correlated with CMR (Fig. 3D).

### Changes in bacterial communities with cultivation

In this study, NMDS analysis and differences at the phylum level indicated that cultivation significantly affected bacterial community composition (Fig. 4A and B). This result is consistent with those of previous studies reporting that cultivation significantly changed microbial diversity and biomass [8-10]. Peatlands contain abundant stores of organic C, N, and P, whereas available N and P fractions mainly depend on groundwater, precipitation, and flow. As a result, N and P are commonly limited in natural peatlands [59]. Similarly, the peatlands of Northeast China are mainly limited by P availability [60]. Those results are consistent with results that bacterial modules #3 and #4 were positively correlated with soil P availability in natural peatlands (Fig. S4A). In addition, cultivation also significantly reduced soil N availability and increased P availability (Table S2). Agricultural fertilization application provides the input of inorganic N and P in soil, but loss of reactive N is severe because of nitrification, denitrification, and leaching with flow [61, 62]. By contrast, P is easily retained by adsorption of soil minerals [63]. In cultivated peatlands, bacterial modules #1 and #3 remained highly positively correlated with P availability (Fig. S4B), indicating that increasing P determines the bacterial community during peatland cultivation. Low soil pH in peatlands is also important factor inhibiting microbial activity, but some microbes have evolved and adapted to such acidic conditions [64]. Similarly, in this study, different bacterial modules also showed different correlations with increasing soil pH in rice-cultivated peatlands (Fig. S4B). In addition, NMDS analysis showed that soil depth also significantly altered bacterial community composition, regardless of cultivation (Fig. 4A). This result is mainly because plant disturbance, hydrologic fluctuations, and oxygen permeability are greater in surface soils than in subsoils, which influences the microbial community [54].

### Keystone taxa associated with carbon mineralization

Identification of keystone bacterial taxa involved in C metabolism can advance the understanding of the changes in microbial communities during C mineralization. In natural peatlands, both *Anaerolineaceae* and *Chloroflexi KD4–96* were keystone taxa associated with bacterial module#2 and were significantly negatively correlated with CMR (Fig. 6A and B). *Anaerolineaceae* are anaerobic bacteria that decompose C via fermentation to generate small molecules such as formate, acetate, hydrogen [65, 66], whereas the *Chloroflexi KD4–96* clade is proposed to be involved in C fixation by assimilating CO_2_ [67]. Furthermore, bacterial module#2 was negatively correlated with carbohydrates, which suggested that those keystone taxa can regulate the bacterial module and contribute to labile C formation. *Defluviicoccus* was the keystone taxon associated with module#3, and it can also degrade many organic compounds [68]. Both *Defluviicoccus* were positively correlated with CMR (Fig. 6C), suggesting an important effect on bacterial module and C decomposition. In cultivated peatlands, *Xanthomonadales* was the keystone taxon associated with bacterial module#2, and it is known to use various C sources and degrade hydrocarbons [69]. Module#2 was also negatively correlated with phenolics, indicating that keystone taxa *Xanthomonadales* could influence the bacterial module structure and functions, causing phenolic degradation. *Arthrobacter* was identified as the keystone taxon for module#3 (Fig. 5D) and it has strong capacity for degrading recalcitrant C [70] while also participating in Fe reduction coupled to C oxidation [71]. Similarly, bacterial module#3 also showed negative correlations with phenolics and carbohydrates (Fig. 5B), which indicate that *Arthrobacter* can regulate the bacterial module and promote labile and recalcitrant C decomposition. *Bacteroidetes vadinHA17* was the keystone taxon influencing module#4, and it can undergo recalcitrant carbon degradation [72]. Here, module#4 only showed negative relations with DOC (Fig. 5B), but phenolics were the dominant components in DOC in cultivated peatlands (Fig. S3B), which imply that *Bacteroidetes vadinHA17* can influence bacterial module and exert positive effects on phenolics decomposition.

**Figure 7:**
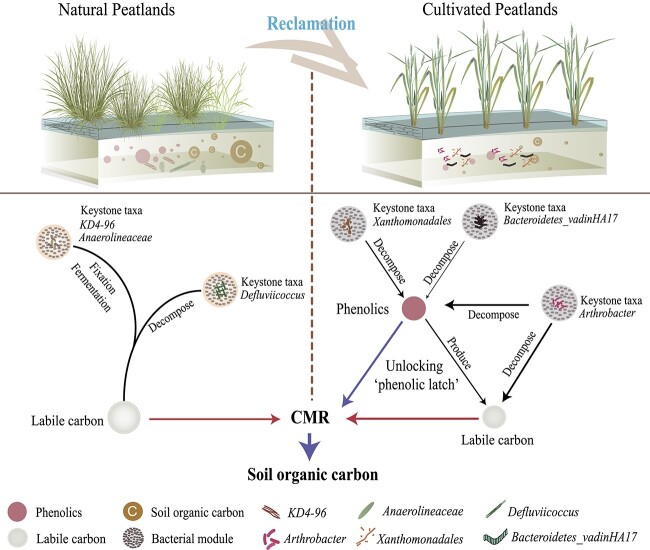
Conceptual model shows that changes of bacterial community remove phenolic constraints on peatland carbon (C) preservation during rice cultivation. Rice cultivation alters bacterial community modules and keystone taxa, whereas in natural peatlands, keystone taxa are involved with fermentation, C fixation, and labile C decomposition, which influence the effects of community modules on C stores and mineralization. In rice-cultivated peatlands, the keystone taxa *Xanthomonadales*, *Arthrobacter*, *and Bacteroidetes_vadinHA17* regulate community modules and promote phenolics and labile C decomposition. Red and blue arrows indicate positive and negative influences, respectively. The width of arrows represents the relative importance.

Microbial keystone taxa often have roles as “ecosystem engineers” and mediate community structure and function [40]. Using co-occurrence diagnosis analysis, subcommunity structure (modules) was analysed to provide insight into community functions. In this study, the keystone taxa in the natural and cultivated systems dominated the bacterial modules and showed different relations with CMR and C factions. In natural peatlands, the keystone bacterial taxa were associated with the functions of fermentation, C fixation, and C decomposition, which associated the community modules with C storage and release. By contrast, in rice cultivated peatlands, the influence of agricultural practices and changes in soil conditions modified the soil bacterial community and keystone taxa. *Xanthomonadales* and *Arthrobacter* were the keystone taxa that could decompose complex C compounds. Specifically, *Xanthomonadales* associated with bacterial modules lead to a negative correlation with phenolics; whereas *Arthrobacter* was associated with the bacterial modules that were negatively correlated with both phenolics and carbohydrates. In addition, phenolics decomposition not only contributes to C mineralization but also produces labile C [30], and the removal of phenolic constraints can further stimulate labile C decomposition [17], thereby accelerating the C decomposition. Collectively, the results suggest a potential pathway in which changes in the bacterial community promote peat C decomposition during rice cultivation by degrading phenolics and removing phenolics constraints on labile C mineralization (Fig. 7). Given that vast reserves of C remain buried in deep soil in rice-converted peatlands, studies on the mechanisms and factors that affect keystone bacterial taxa in the microbiome may provide keys to mitigating their C emissions and promoting sustainable peatland management and contributing to climate change mitigation.

## Supplementary Material

Supplementary_ycae022

## Data Availability

All sequences have been deposited in the National Center for Biotechnology Information (NCBI) Sequence Read Archive (SRA) under the BioProject number PRJNA1002482. The original data and related code are available online in the Figshare data repository (https://doi.org/10.6084/m9.figshare.24919758).
