# Supplementary material for: Changes in bacterial communities during rice cultivation remove phenolic constraints on peatland carbon preservation
Source: ISME Commun. 2024 Feb 6;4(1):ycae022. doi: 10.1093/ismeco/ycae022 (PMC10945358; doi:10.1093/ismeco/ycae022)
Supplement: Supplementary_ycae022 [file supplementary_ycae022.docx]

**Running Title: Cultivation and Peat Carbon Loss**

**Changes in bacterial communities during rice cultivation remove phenolic constraints on peatland carbon preservation**

^1^State Key Laboratory of Black Soils Conservation and Utilization, Key Laboratory of Wetland Ecology and Environment, Heilongjiang Xingkai Lake Wetland Ecosystem National Observation and Research Station, Northeast Institute of Geography and Agroecology, Chinese Academy of Sciences, Changchun 130102, China.

^2^College of Forestry and Grassland, Jilin Agriculture University, Changchun 130118, China

^3^School of Natural Sciences, Bangor University, Bangor LL57 2UW, UK.

^*^Corresponding author:

Yuanchun Zou, email address: zouyc@iga.ac.cn; physical address: 4888 Shengbei Street, Changchun City, Jilin Province, China

^**^Corresponding author:

Ming Jiang, email address: jiangm@iga.ac.cn; physical address: 4888 Shengbei Street, Changchun City, Jilin Province, China

**Contents of this file**

Tables S1–S5

Figures S1–S4

**Supplemental Tables**

**Table S1**. Soil properties in Jinchuan (JC), Sipeng (SP), and Yushugou (YSJ) peatlands. TN, total nitrogen; TP, total phosphorus; AN, available N; AP, available P.

| Variable | Depth  (cm) | JC | | SP | | YSJ | |
| --- | --- | --- | --- | --- | --- | --- | --- |
|  |  | Peatlands | Cultivation | Peatlands | Cultivation | Peatlands | Cultivation |
| TN  (%) | 0–15 | 18.2±0.46 | 7.0±0.65 | 18.3±0.30 | 5.4±0.40 | 19.6±0.43 | 7.8±0.41 |
|  | 15–30 | 17.5±0.13 | 15.4±1.39 | 17.5±1.13 | 16.4±1.33 | 18.0±0.94 | 7.2±0.56 |
| AN  (mg/kg) | 0–15 | 155.9±17.69 | 14.6±1.80 | 31.9±7.11 | 13.5±0.42 | 55.7±4.68 | 38.5±3.75 |
|  | 15–30 | 49.7±2.00 | 24.2±2.56 | 34.0±1.79 | 23.8±2.19 | 14.4±1.52 | 39.9± 8.47 |
| TP  (g/kg) | 0–15 | 0.7±0.02 | 1.5±0.10 | 1.0±0.04 | 1.0±0.02 | 1.3± 0.05 | 2.4±0.15 |
|  | 15–30 | 0.6±0.02 | 1.0±0.11 | 0.8±0.04 | 0.9±0.03 | 1.4±0.07 | 2.2±0.12 |
| AP  (mg/kg) | 0–15 | 21.6±0.44 | 48.4±8.34 | 16.3±0.57 | 60.1±1.21 | 14.6±2.22 | 85.9±7.73 |
|  | 15–30 | 5.7±0.19 | 18.6±4.58 | 10.9±1.37 | 25.0±4.87 | 8.5±1.21 | 77.9±4.97 |
| pH | 0–15 | 6.0±0.02 | 6.2±0.02 | 5.5±0.17 | 6.0±0.03 | 5.4±0.01 | 5.5±0.10 |
|  | 15–30 | 5.6±0.09 | 6.1±0.09 | 5.6±0.17 | 6.1±0.02 | 5.5±0.04 | 5.5±0.15 |

**Table S2.** Effects of cultivation and depth on soil properties across Jichuan (JC), Sipeng (SP), and Yushugou (YSJ) peatlands. TN, total nitrogen; TP, total phosphorus; AN, available N; AP, available P. Different letters indicate significant differences (*p* < 0.05).

| **Variable** | **Natural peatlands** | | **Cultivated peatlands** | |
| --- | --- | --- | --- | --- |
|  | 0–15 cm | 15–30 cm | 0–15 cm | 15–30 cm |
| TN (g/kg) | 18.70±0.28a | 17.66±0.45a | 6.73±0.39c | 13.01±1.38b |
| AN (mg/kg) | 81.21±17.25a | 32.74±4.46b | 22.19±3.70b | 29.28±3.56b |
| TP (g/kg) | 0.99±0.08b | 0.93±0.10b | 1.63±0.18a | 1.38±0.19a |
| AP (mg/kg) | 17.49±1.14c | 8.36±0.85c | 64.80±5.84a | 40.50±8.39b |
| pH | 5.63±0.12bc | 5.56±0.06c | 5.91±0.10a | 5.88±0.10ab |

**Table S3.** Effects of cultivation and depth on bacterial phyla across Jichuan (JC), Sipeng (SP), and Yushugou (YSJ) peatlands. Different letters indicate significant differences (*p* < 0.05).

| **Variable** | **Natural peatlands** | | **Cultivated peatlands** | |
| --- | --- | --- | --- | --- |
|  | 0–15 cm | 15–30 cm | 0–15 cm | 15–30 cm |
| *Acidobacteria* | 14.47±0.75a | 13.50±0.41ab | 11.98±0.71b | 12.61±1.27ab |
| *Actinobacteria* | 13.54±0.73a | 11.37±0.39b | 12.08±0.51ab | 12.31±0.51ab |
| *Alphaproteobacteria* | 16.06±0.56a | 9.37±0.46b | 9.28±1.19b | 7.78±1.10b |
| *Bacteroidetes* | 2.67±0.18c | 3.64±00.26b | 5.26±0.24a | 3.48±0.33bc |
| *Betaproteobacteria* | 10.03±0.58b | 6.40±0.29c | 15.40±0.92a | 8.82±0.70b |
| *Chlorobi* | 2.29±0.12c | 3.35±0.09a | 2.77±0.17bc | 2.97±0.23ab |
| *Chloroflexi* | 11.76±0.60c | 20.77±0.37ab | 15.16±0.99c | 24.41±2.58a |
| *Deltaproteobacteria* | 7.74±0.36c | 9.02±0.31bc | 10.54±0.48a | 7.92±0.50c |
| *Verrucomicrobia* | 4.44±0.37b | 6.16±0.30a | 3.02±0.12c | 4.08±0.30b |
| *Gammaproteobacteria* | 4.66±1.61a | 1.39±0.15b | 2.28±0.31ab | 1.60±0.15b |

**Table S4.** Topological properties of co-occurring bacterial networks obtained in natural and cultivated peatlands.

| Network metrics | Bacterial community | |
| --- | --- | --- |
|  | Types | |
|  | Natural peatlands | Cultivated peatlands |
| **Empirical networks** |  |  |
| Number of nodes | 1492 | 1522 |
| Number of edges | 37770 | 39892 |
| Number of positive correlations | 23969 | 25320 |
| Number of negative correlations | 13801 | 14572 |
| Average path length | 2.871 | 2.854 |
| Graph density | 0.034 | 0.034 |
| Average clustering coefficient | 0.569 | 0.534 |
| Number of modules | 4 | 4 |

**Table S5.** Keystone bacterial taxa in natural and cultivated peatlands. Gene significance (GS) indicates the association of genes with environmental traits; module membership (MM) indicates gene-to-module association; and hub genes (keystone taxa) are defined as those that were significant for both GS and MM at 0.05. Values are correlation coefficients. **p* < 0.05; ***p* < 0.01; ****p* < 0.001. OTU, operational taxonomic unit.

| **Modules** | **Natural peatlands** | | | | |
| --- | --- | --- | --- | --- | --- |
|  | **OTU ID** | **Phylum/Class/Order**  **/Family** | **Affiliation** | **GS** | **MM** |
| **Module#2** | **OTU6** | *Anaerolineales* | *Anaerolineaceae* | −0.86^***^ | 0.88^***^ |
|  | **OTU43** | *Anaerolineales* | *Anaerolineaceae* | −0.81^***^ | 0.87^***^ |
|  | **OTU235** | *Anaerolineales* | *Anaerolineaceae* | −0.73^***^ | 0.84^***^ |
|  | **OTU402** | *Anaerolineales* | *Anaerolineaceae* | −0.57^**^ | 0.77^***^ |
|  | **OTU67** | *Chloroflexi* | ***KD4-96*** | −0.54^**^ | 0.72^***^ |
|  | **OTU30** | *Chloroflexi* | ***KD4-96*** | −0.59^**^ | 0.89^***^ |
| **Module#4** | **OTU487** | *Rhodospirillaceae* | *Defluviicoccus* | 0.44^*^ | 0.91^***^ |
|  | **Cultivated peatlands** | | | | |
| **Module#2** | **OTU1132** | *Gammaproteobacteria* | *Xanthomonadales* | 0.75^***^ | 0.88^***^ |
| **Module#3** | **OTU33** | *Micrococcaceae* | *Arthrobacter* | 0.44^*^ | 0.87^***^ |
| **Module#4** | **OTU1316** | *Bacteroidetes* | *Bacteroidetes_vadinHA17* | 0.53^**^ | 0.56^**^ |
|  | **OTU1372** | *Bacteroidetes* | *Bacteroidetes_vadinHA17* | 0.70^***^ | 0.54^**^ |

**Supplemental Figures**

**Fig. S1.** Mixed models of the effects of cultivation on bacterial alpha diversity during peatlands cultivation. (a) Richness; (b) Shannon diversity. Research site was the random factor, *Rm*^2^ was the variance explained by the fixed effect, and *Rc*^2^ was the variance explained by both fixed and random effects. Values are the mean ± SE, *n* = 12.

**Fig. S2.** Bacterial co-occurrence network in natural and cultivated peatlands. Circles indicate the nodes and different colors represent the different modules. (a) Natural peatlands; (b) cultivated peatlands.

**Fig. S3.** Relations between environmental variables and oligotrophs to copiotrophs ratio. Relations between dissolved organic carbon (DOC), phenolics, available phosphorus (AP), and available nitrogen (AN) and oligotrophs to copiotrophs ratio in (a-d) natural and (e-h) cultivated peatlands. Solid lines indicate significant linear correlations.

**Fig. S4.** Pearson correlation coefficients for module eigengenes and environmental variables in (a) natural and (b) cultivated peatlands. ^*^*p* < 0.05; ^**^*p* < 0.01; ^***^*p* < 0.001.

**Figure S1**


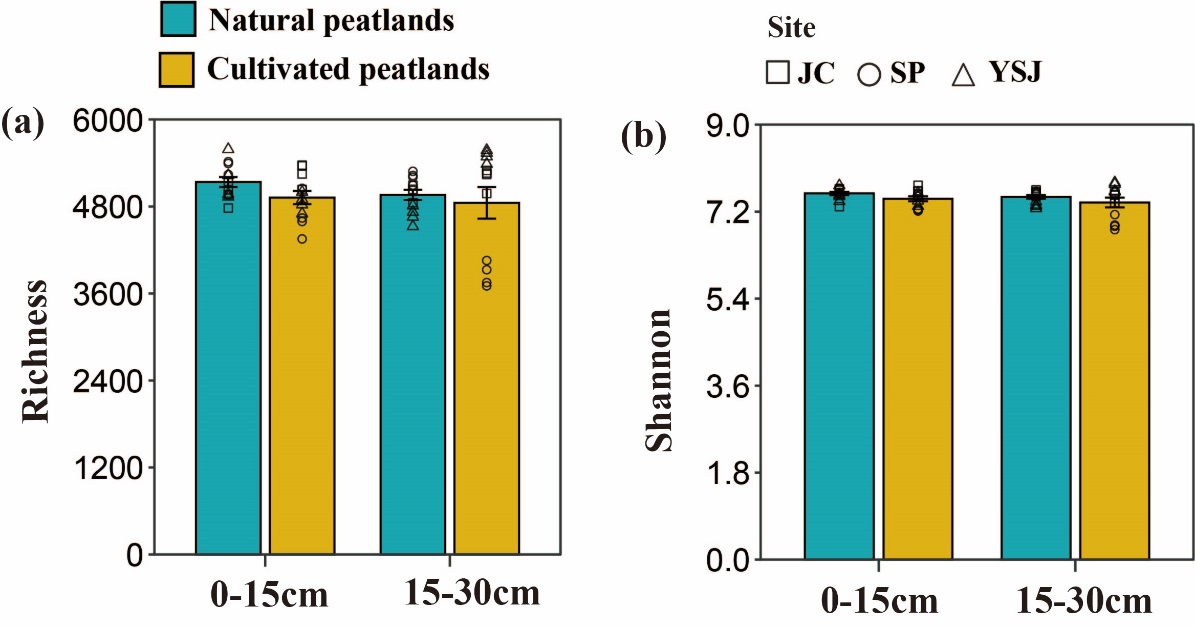


**Figure S2**

**
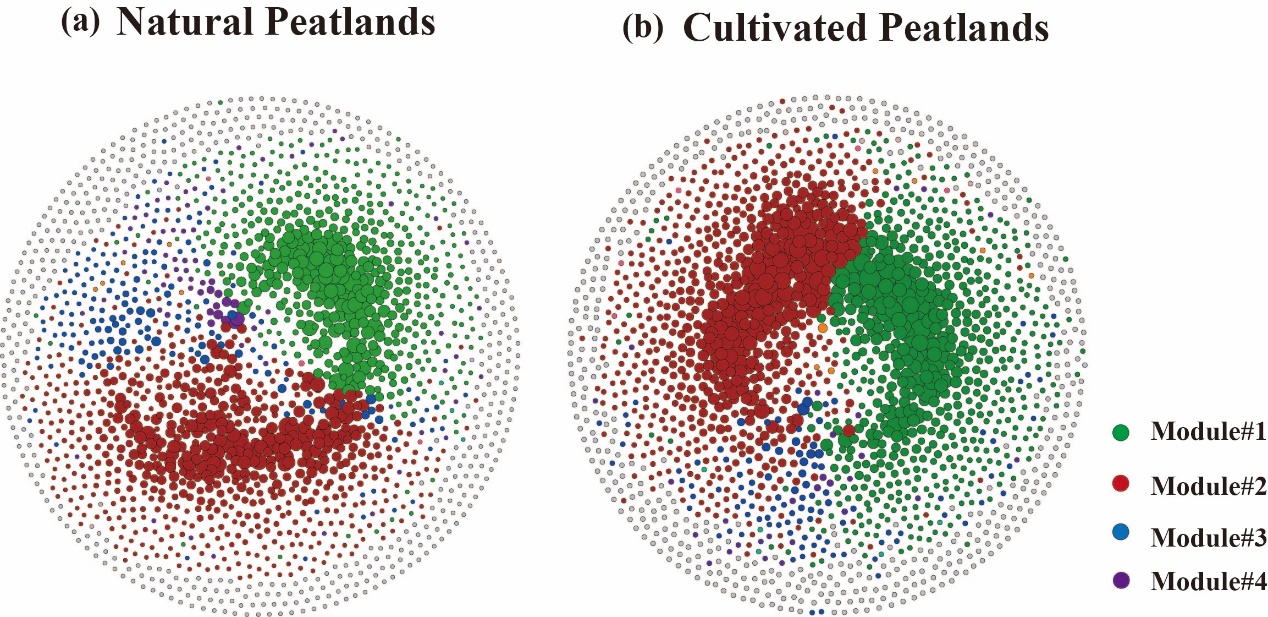
**

**Figure S3**


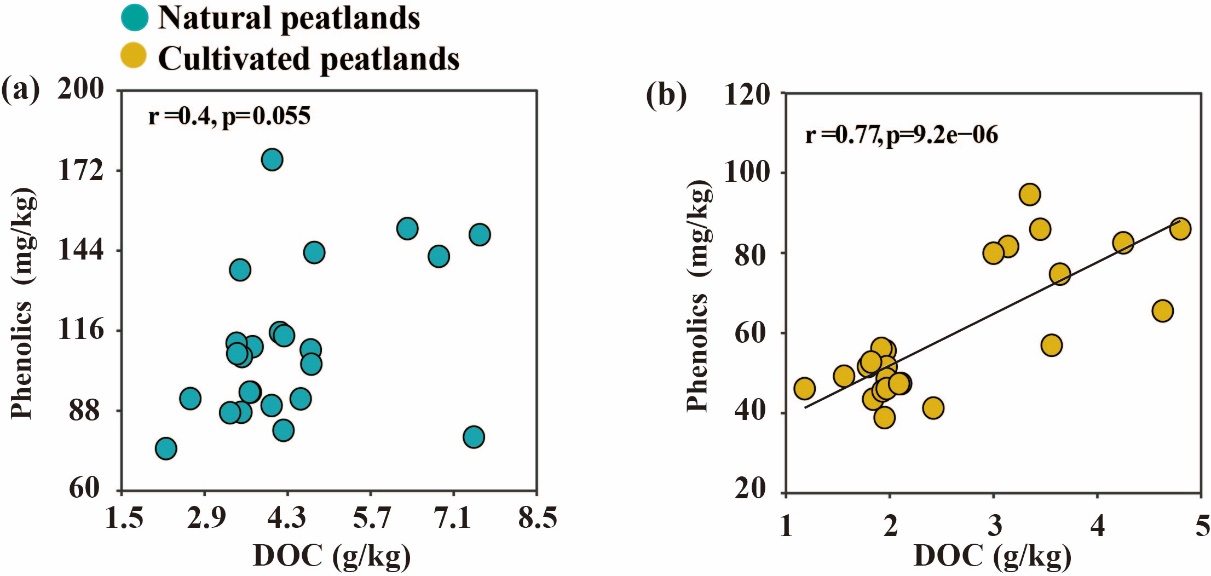


**Figure S4**

**
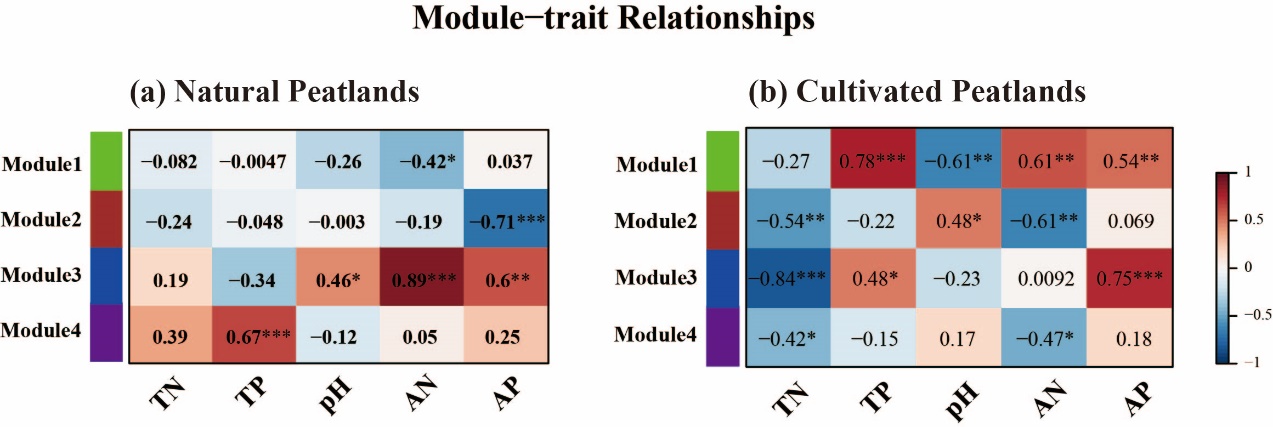
**
